# Renal hyperparathyroidism- a risk factor in the development of encapsulating peritoneal sclerosis

**DOI:** 10.3389/fendo.2024.1282925

**Published:** 2024-03-19

**Authors:** Zia Moinuddin, Kelvin Wang, Catherine Fullwood, Elizabeth Wiredu, Alastair Hutchison, Anand Vardhan, Sarah E. Herrick, Angela Summers, Titus Augustine, David van Dellen

**Affiliations:** ^1^ Department of Renal and Pancreas Transplantation, Manchester Royal Infirmary (National Commissioning Group (NCG) funded United Kingdom Referral Centre for EPS Surgery), Manchester, United Kingdom; ^2^ School of Biological Sciences, Faculty of Biology, Medicine and Health, The University of Manchester, Manchester, United Kingdom; ^3^ Department of Statistics, Research and Innovation, Manchester University NHS Foundation Trust, Manchester, United Kingdom; ^4^ Centre for Biostatistics, University of Manchester, Manchester, United Kingdom; ^5^ Medical Statistics, Data Solution Services, Liverpool, United Kingdom; ^6^ Department of Nephrology, Manchester Royal Infirmary, Manchester, United Kingdom

**Keywords:** EPS, CKD-MBD, peritoneal dialysis, calcification, peritoneal fibrosis

## Abstract

**Background:**

Encapsulating peritoneal sclerosis (EPS) is a rare complication of prolonged peritoneal dialysis (PD) exposure, characterised by peritoneal thickening, calcification, and fibrosis ultimately presenting with life-threatening bowel obstruction. The presence or role of peritoneal calcification in the pathogenesis of EPS is poorly characterised. We hypothesise that significantly aberrant bone mineral metabolism in patients on PD can cause peritoneal calcification which may trigger the development of EPS. We compared the temporal evolution of bone mineral markers during PD in EPS patients with non-EPS long-term PD controls.

**Methods:**

Linear mixed model and logistic regression analysis were used to compare four-monthly serum levels of calcium, phosphate, parathyroid hormone, and alkaline phosphatase (ALP) over the duration of PD exposure in 46 EPS and 46 controls (PD, non-EPS) patients.

**Results:**

EPS patients had higher mean calcium (2.51 vs. 2.41 mmol/L) and ALP (248.00 vs. 111.13 IU/L) levels compared with controls (p=0.01 and p<0.001 respectively, maximum likelihood estimation). Logistic regression analysis demonstrated that high serum calcium and phosphate levels during PD were associated with a 4.5 and 2.9 fold increase in the risk of developing EPS respectively.

**Conclusion:**

High levels of calcium and phosphate in patients on PD were identified to be risk factors for EPS development. Possible reasons for this may be an imbalance of pro-calcifying factors and calcification inhibitors promoting peritoneal calcification which increases peritoneal stiffness. Mechanical alterations may trigger, unregulated fibrosis and subsequent development of EPS. Improved management of secondary hyperparathyroidism during PD may ultimately diminish the EPS risk.

## Introduction

Encapsulating Peritoneal Sclerosis (EPS) is a rare and often fatal complication of long-term peritoneal dialysis (PD), characterised by the development of a fibrous cocoon enveloping the intestines ultimately resulting in fulminant bowel obstruction (1). Prolonged PD therapy is the most important risk factor in the development of EPS (2–4). The widely accepted theory for the pathogenesis of EPS is explained by the *two-hit fibrosis model* (3, 4). Uraemia, exposure to PD and the consequent inflammation result in chronic repeated serosal damage and repair, ultimately leading to peritoneal thickening and sclerosis. This, if followed by a second insult (including recurrent peritonitis, transplantation, sudden cessation of PD, peritoneal exposure to disinfectants, coupled with a genetic predisposition), can lead to uncontrolled fibrosis and EPS development (4–10). Bowel tethering and dilatation, peritoneal thickening, and peritoneal calcification are considered to be diagnostic signs of EPS on computed tomography (CT) imaging (4, 11–13). Furthermore, although peritoneal calcification has been described in patients on PD without EPS, its presence is found more often in EPS patients (11, 14, 15). However, the role of peritoneal calcification in EPS development or PD membrane failure has not been investigated, in part due to the relative rarity of the disease, which has resulted in clinical studies remaining a challenge to perform.

Secondary hyperparathyroidism (SHPT) is a common problem in patients with chronic kidney disease (CKD) especially in those requiring dialysis (16). Progressive decline in renal function leads to impaired phosphate excretion and reduced synthesis of calcitriol, the active form of vitamin D (17). The resultant dysregulation of calcium and phosphate homeostasis leads to increased levels of serum phosphate and fibroblast growth factor 23 (FGF-23) (17). These changes result in increased synthesis and secretion of parathyroid hormone (PTH) and parathyroid hyperplasia, contributing to the development of the characteristic biochemical features of chronic kidney disease-mineral bone disorder (CKD-MBD): high phosphate, high PTH, low calcium and vitamin D deficiency (17). Additionally, the enzyme alkaline phosphatase (ALP), is often elevated due to the effects of PTH and high bone turnover. It is known that such altered bone mineral metabolism and secondary hyperparathyroidism are associated with vascular calcification and increased mortality risk in end stage renal failure (ESRF) patients on dialysis (17, 18). However, there is a paucity of data describing the development and subsequent impact of peritoneal calcification in patients on PD and whether it occurs due to the same molecular mechanism of vascular calcification. Indeed limited case reports and small retrospective case series have provided conflicting information regarding the role of secondary hyperparathyroidism and altered bone mineral metabolism in subsequent development of peritoneal calcification (19–22). Similarly, the role of secondary hyperparathyroidism in the evolution of EPS has not been described. We hypothesise that patients who develop EPS have poorly controlled secondary hyperparathyroidism initially, with significantly altered bone mineral metabolism. The aim of this study was to compare the temporal evolution of markers of bone mineral metabolism during PD in EPS and non-EPS patients.

## Methods

### Study design

A retrospective cohort study was designed to compare bone mineral metabolism between EPS patients and a control group of patients on long-term PD without EPS. The study was approved by the Research and Development (R&D) department at Manchester University Hospital NHS Foundation Trust (MFT) (R04141). Research Ethics Committee review was not required under the Governance Arrangements for Research Ethics Committees (GAfREC) guidelines for research limited to the use of previously collected information.

### Inclusion/exclusion criteria and data collection

Inclusion criteria of the EPS group: adult patients (over 18 years of age) with a diagnosis of EPS made at our institution between 2000 and 2013. EPS cases were diagnosed on clinical suspicion, and subsequently confirmed by CT scan or at the time of surgery (diagnostic laparoscopy and/or laparotomy). These patients were identified using a contemporaneously maintained database at our institution, a nationally commissioned EPS international referral service.

Inclusion criteria of the control group: long term (>4 years exposure) PD patients (over 18 years of age) from local units between 2003 and 2013. These patients had not developed EPS at the time of data collection.

Exclusion criteria: patients were excluded from either group if they were under the age of 18 at PD commencement, or if there was an active diagnosis of cancer, or other endocrine or metabolic condition confounding calcium homeostasis. EPS cases of non-PD aetiology were excluded from the disease cohort.

Demographic data (including age, gender, ethnicity) as well as PD relevant (cause of ESRF, date of PD start and cessation, and relevant medication) history were collated. Data relevant to calcium regulation were also monitored [parathyroidectomy history, serum corrected calcium (Ca), phosphate (P), alkaline phosphatase (ALP), calcium phosphate product (Ca*P) and parathyroid hormone (PTH) levels] at 4 monthly intervals from the time of onset of PD until cessation or 5 years from therapy initiation. Post-parathyroidectomy PTH measurements were excluded from analysis (unless there was a subsequent recurrent rise in PTH suggestive of ongoing parathyroid activity).

### Statistical analysis

A linear mixed-effects model was used to compare the temporal change in the levels of each dependent variable (Ca/P/PTH/ALP/Ca*P), from PD onset until cessation, between the EPS and control groups. This model was chosen due to the presence of asymmetric data as a result of varied PD exposure duration, missing data and dependence of repeated measures across time points. Dependent variable changes were analysed by comparing the average slope of the regression line over the duration of PD between the EPS and control groups to test for statistical significance. The intercept and slope of the regression were allowed to vary between subjects through the inclusion of random effects. In the model, the disease groups (EPS and control) and the change in levels of the dependent variables in the groups at each time point across the duration of PD were introduced as fixed effects. The potential impact of the other variables on the dependent variable was mitigated by introducing them into the model as random effects. P-values were obtained by maximal likelihood ratio estimations of the full model with the fixed effect in question against the model without the fixed effect in question. The linear mixed-effects model provides unbiased estimates of effect with the assumption that missing data is randomly omitted and independent of outcome.

Multivariate logistic regression analysis was also used to compare the mean serum levels of corrected calcium, phosphate, alkaline phosphatase and median parathyroid hormone levels over the entire duration of PD across the two groups. The National Kidney Foundation’s Kidney disease outcome quality initiative (NKF-KDOQI) and Kidney Disease Improving Global Outcome (KDIGO) recommendations for management of chronic kidney disease- mineral and bone disorder (CKD-MBD) (23) guided cut-off values for each dependent variable for the logistic regression analysis [serum calcium (2.54 mmol/l), serum phosphate (1.78mmol/l), serum alkaline phosphatase (104 IU/L) and serum PTH [<120pg/ml, 120-540 pg/ml and >540pg/ml)].

The incidence of hypercalcaemia (serum calcium levels >2.65 mmol/l) at various time points during PD was compared between the two groups using Fisher’s exact test. Mann-Whitney and Fisher’s exact/Chi-square tests were used to compare the baseline characteristics (ethnicity, gender, age, duration of PD, medication history, parathyroidectomy) between the two groups. Data were presented as mean [standard error of the mean (SEM)] or median [interquartile range (IQR)] values. Differences were accepted as statistically significant at p<0.05. SPSS version 22 (IBM, New York, USA) was used to perform logistic regression and mixed model analysis. GraphPad Prism 6.04 Software (GraphPad Software Inc., USA) was used to perform Mann-Whitney tests, Fisher’s exact tests, Chi-Square tests and generation of graphs.

## Results

### Demographic and clinical characteristics

**Figure 1:**
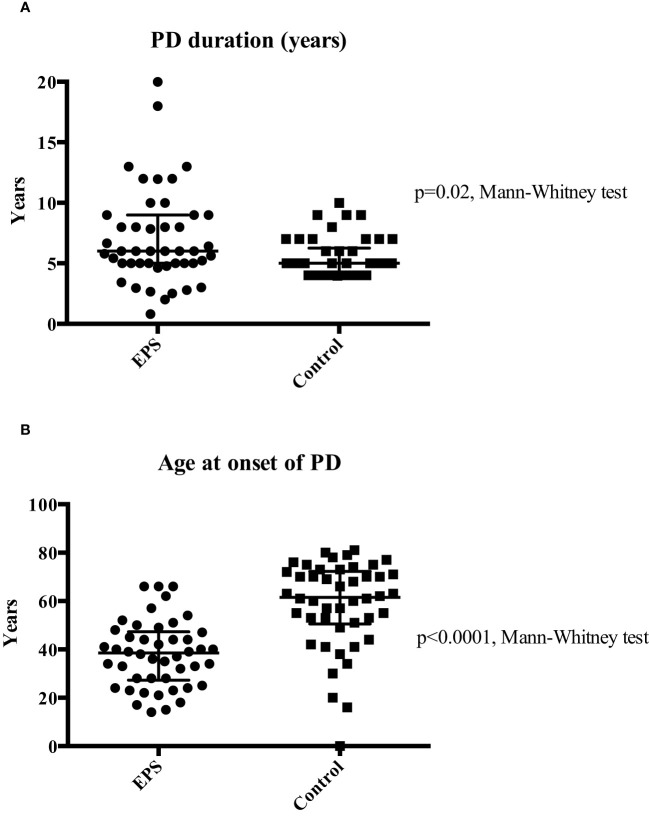
Comparison of median PD duration (Panel **A**) and median age at PD onset (Panel **B**) between the two groups. Patients in the EPS group were exposed to a significantly longer duration of PD (6 vs. 5 years; p=0.02 Mann-Whitney test). Extensive variation in duration of PD at both ends of the spectrum was noticed in the EPS group. Patients in the EPS group were significantly younger than patients in the control group (38.5 vs. 61.5 years) (p<0.01, Mann-Whitney test).

The study identified a total of 92 patients, consisting of 46 patients included in the EPS (disease) group and 46 controls who were treated with PD for four years or longer and matched for demographic and disease data. The latter were selected from a database of 175 patients from local centres. The demographic and clinical characteristics of both groups were assessed (**Table 1**). The predominant differences between the groups were related to PD duration and age at onset, calcium-based phosphate binders and vitamin D therapy, and cinacalcet therapy. The median age of patients at the onset of PD was significantly higher in the control group (p<0.01, Mann-Whitney Test). EPS patients also had a longer median PD duration compared to the control group (p=0.02, Mann-Whitney Test) although there was noticeably wider variation in the former group (**Figure 1**). More control patients (46) were treated with calcium based phosphate binders and vitamin D during the course of PD when compared to the EPS group (38) (p=0.01, Fisher’s exact test). There was a trend towards significance with more control patients (7, 15.2%) receiving cinacalcet therapy compared to the EPS group (1, 2.2%) (p=0.06, Fisher’s exact test). Other demographic features were non-significant on comparison.

**Table 1:** Demographic and clinical characteristics of study subjects in the two groups (Encapsulating Peritoneal Sclerosis (EPS) vs. Control).

Characteristics	EPS Group (N=46)	Control group (N=46)	p- value*
Peritoneal Dialysis History			
Age at PD onset (years)	38.5 (27.0-47.0)	61.5 (50.5-72.3)	**<0.01^¶^**
PD duration (years)	6.0 (5.0-9.0)	5.0 (4.0-6.3)	**0.02^¶^**
**Demographic Data**			
Ethnicity: Caucasian	38 (82.6)	35 (76.1)	0.61^*^
Sex: Male	22 (47.1)	29 (63.0)	0.21^*^
Therapeutic History			
Calcium based phosphate binders and Vitamin D therapy	38 (82.6%)	46 (100.0)	0.01^*^
Cinacalcet therapy	1 (2.2%)	7 (15.2)	0.06^*^
PD peritonitis	21(4.7)	27(58.7)	0.30^*^
Parathyroidectomy	18 (39.1)	10 (21.7)	0.12^*^
Warfarin therapy	1(2.2)	1(2.2)	1.0^*^
**Medical History**			
Diabetes	5 (10.9)	11 (23.9)	0.17^*^
Renal transplant post-PD	11 (23.9)	9 (19.6)	0.80^*^
**Cause of Renal Failure**			0.07^**^
Hypertension	3 (6.5)	8 (17.4)	
Diabetes	3 (6.5)	9 (19.6)	
Reflux Nephropathy	8 (17.4)	4 (8.7)	
IgA Nephropathy	10 (21.7)	5 (10.9)	
Cystic	9 (19.6)	9 (19.6)	
Congenital	7 (15.2)	2 (4.4)	
Unknown	6 (13%)	9 (19.6)	

Data in the ‘Peritoneal Dialysis History’ section are reported as ‘Median (IQR)’; data in the remaining sections are expressed as ‘Number (%)’. P-values are based on ^*^Fisher’s exact and ^**^Pearson’s Chi-square test for categorical variables and ^¶^Mann-Whitney test for continuous variables. PD (peritoneal dialysis).

Numbers in bold denote a statistically significant p- value.

### Serum calcium

**Figure 2:**
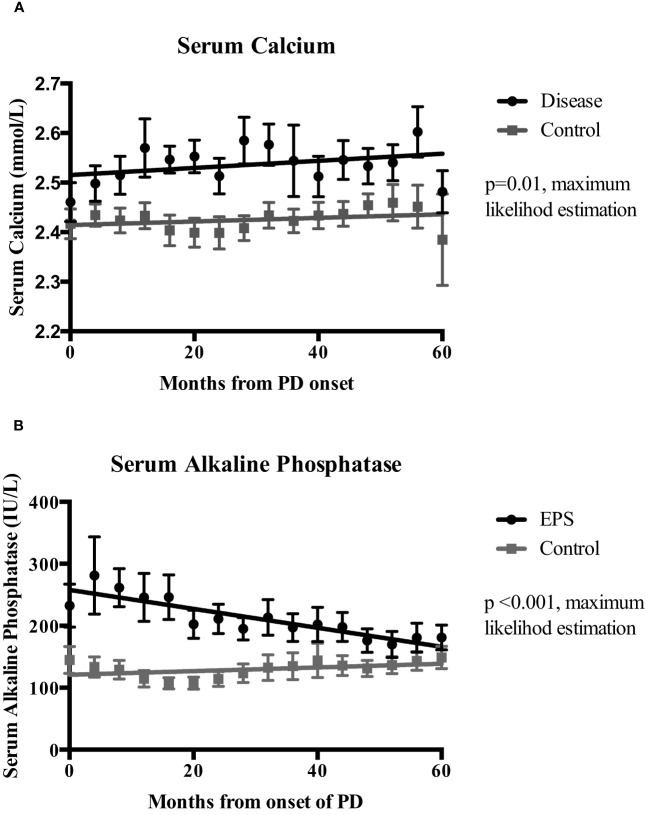
Mixed-effects analysis of the temporal change of serum calcium (Ca)(Panel **A**) and serum alkaline phosphatase (ALP) (Panel **B**) levels between the EPS and control groups over the duration of peritoneal dialysis (PD). Analysis used dependent variable (Ca/ALP), group, time and, group-by-time variables. EPS patients had significantly higher mean serum calcium (2.51 vs. 2.41 mmol/l; p=0.01, maximum likelihood estimation (MLE) and alkaline phosphatase levels (248 vs. 11.13 IU/L; p<0.01, MLE) during PD when compared to the control group. I bars represent standard error of the mean (SEM).

**Figure 3:**
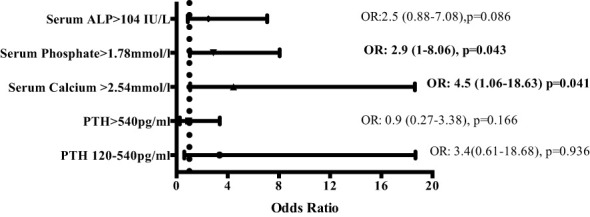
Multivariate logistic regression analysis depicting odds ratios of risk of developing EPS depending on serum levels of markers of bone mineral metabolism (odds ratios [OR] with 95% confidence intervals). Serum calcium levels of >2.54mmol/l and serum phosphate levels of >1.78 mmol/l increase the risk of developing EPS by 4.5 and 2.9-fold respectively. (ALP – alkaline phosphatase; PTH – parathyroid hormone).

**Figure 4:**
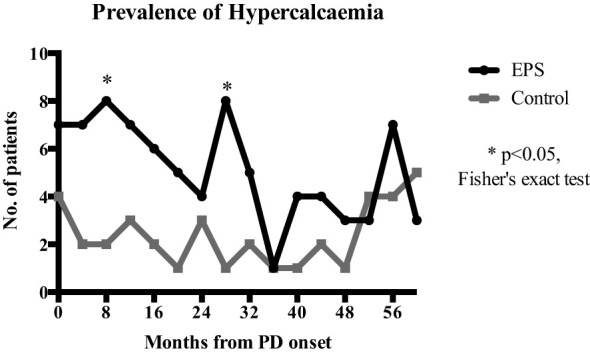
Comparison of hypercalcaemia frequency (serum calcium >2.65mmol/l) at each 4-monthly time point over PD duration. EPS patients had a higher frequency of hypercalcaemia compared to the control group with statistical significance at 8 (p=0.045) and 28 months (p=0.015) (Fisher’s exact test).

Linear mixed-effects analysis that incorporated the independent variables of study groups, PD duration (time), and group-by-time interactions demonstrated significantly higher serum calcium levels in the EPS group compared with controls [2.51 (0.03) vs. 2.41 (0.04) mmol/l; p=0.01, maximum likelihood estimation] (**Figure 2**). There was an overall increase in the calcium levels, but no significant difference in the rate of change between the two groups over the duration of PD (**Figure 2**). Multivariate logistic regression analyses demonstrated that patients with serum calcium levels of more than 2.54 mmol/l have a 4.5-fold increase in probability of subsequent EPS development [OR: 4.5 (1.06-18.63), p=0.04] (**Figure 3**). Analysis of the frequency of hypercalcaemia (Serum calcium >2.65 mmol/l) at each time point of data collection during PD showed a higher frequency in the EPS group when compared to controls. However, statistically significant differences were only seen at 8 months and 28 months from PD onset (Fisher’s exact test) (**Figure 4**).

### Serum alkaline phosphatase

Linear mixed-effects analysis showed that serum ALP levels were significantly higher in the EPS group compared to controls [248 (22.12) vs. 111.13 (30.81) IU/L; p <0.01, maximum likelihood estimation] (**Figure 2**). ALP levels declined over the course of PD in the EPS group in comparison to controls (p=0.01, maximum likelihood estimation). However, despite this, the mean ALP level at the end of the period under study was still higher in the EPS group. Multivariate logistic regression analysis demonstrated that patients with serum ALP levels greater than 104 mmol/l had a 2.5-fold increase in likelihood of subsequent EPS development. However, this only approached statistical significance [OR: 2.5 (0.88-7.08), p=0.09] (**Figure 3**).

### Serum phosphate

Hyperphosphataemia was prevalent across both groups with no significant change in levels of serum phosphate over time [EPS vs. control; 1.65 (0.06) vs. 1.63 (0.08) mmol/l; p=0.80, maximum likelihood estimation]. Multivariate regression analysis showed that serum phosphate levels of greater than 1.78 mmol/l conferred a 2.9-fold increase in the likelihood of EPS development [OR: 2.9 (1-8.06), p=0.04] (**Figure 3**).

### Serum parathyroid hormone and calcium phosphate product

There was no significant difference in the levels of parathyroid hormone over the duration of PD across both groups [EPS vs. control; 266.48 (54.34) vs. 200.19 (75.89) pg/ml; p=0.38, maximum likelihood estimation]. However, EPS patients had a higher Ca*P value [4.07 (0.16) vs. 3.90 (0.22) mmol^2^/l^2^], although this was not statistically significant (p= 0.46, maximum likelihood estimation). In addition, the Ca*P of 4.07 mmol^2^/l^2^ was above the NKF-KDOQI referenced threshold of 4 mmol^2^/l^2^ (24).

## Discussion

This cohort study is the first to provide an insight into the temporal evolution of bone mineral markers in serum during PD and their association with subsequent EPS development. Our results demonstrate significantly and chronically elevated serum corrected calcium and ALP levels in EPS patients compared to non-EPS patients on long-term PD. High serum calcium and phosphate levels were associated with a 4.5 and 2.9-fold increase in risk for EPS development, respectively. Higher levels of Ca*P, beyond the NKF-KDOQI recommended threshold of 4 mmol^2^/l^2^, were also found in the EPS group when compared to controls which, although not statistically significant, possibly represents an increased risk factor for ectopic peritoneal calcification. In addition, a significantly higher proportion of control patients received treatment for secondary hyperparathyroidism (phosphate binders and vitamin D analogues). This suggests that secondary hyperparathyroidism was more optimally managed in the control group. Uncontrolled secondary hyperparathyroidism can therefore be postulated to be a potential risk factor in the development of EPS. This has significant implications in terms of potential therapeutic opportunities for PD patients to ameliorate the risk of subsequent EPS development. This also has obvious implications for a disease process that ultimately has the potential for catastrophic complications, which include fulminant intestinal obstruction and death.

The previous findings of other groups support our conclusion that secondary hyperparathyroidism alone can represent a significant risk factor for EPS development in PD patients; in a retrospective analysis of 58 EPS patients, Tseng et al. (24) found that, in combination with several other factors, serum PTH levels greater than 384 pg/ml was predictive of severe EPS. The other factors in this paper (EPS diagnosis after PD discontinuation, bowel tethering on CT, bloody ascites, and CRP level ≥29 mg/L) were not related to bone mineral metabolism, however. Alatab et al. (25) reported higher mean PTH levels in EPS patients when compared to a non-EPS cohort (178 pg/ml vs 118 pg/ml), although this did not reach statistical significance. Of note, this could have been due to a small EPS cohort consisting of only 12 cases. Similar non-significant findings of elevated PTH levels in EPS cases were reported in another 2022 retrospective study (26). Once again, this study was limited by the inclusion of only a small number (n=16) of EPS diagnoses.

### Limitations and future work

Although, the patients in the EPS group had a longer median duration of PD exposure by one year, this difference needs to be interpreted with caution due to the wide variation in PD duration in the EPS group. The median age of the EPS group was 14 years younger. This may represent a confounding factor, as younger patients have been reported to be at increased risk of EPS, possibly due to improved ability for regeneration and repair (7, 8, 27). In addition, older patients are less likely to have a long PD exposure, which could account for the reduced EPS incidence due to reduced cumulative risk (3). The control group patients may still develop EPS, but all have had PD exposure of at least 4 years along with up-to-date follow up with no evidence of signs or symptoms of the disease to the point of reporting. Advancing age can also have an effect on the serum levels of bone minerals and is a risk factor for the development of secondary hyperparathyroidism (28, 29). However, as our entire study group is composed of ESRF patients with prevalent secondary hyperparathyroidism, the effect of age on bone minerals may not be relevant to this population. Nevertheless, the difference in age in between the groups is a potential confounding factor that requires future investigation.

It is acknowledged that this is a retrospective study, and therefore has the potential to be affected by under-powering, selection and confounding bias. However, the nature and rarity of EPS reflects that the patient cohort selected as well as the numbers recruited actually represent a sizeable and representative population. There are a number of other potential factors integral to bone mineral metabolism and ectopic calcification, including vitamin D, magnesium (30), fibroblast growth factor-23 (FGF-23), and fetuin-A (31). The retrospective nature of data collection, precluded analysis of these as they were not routinely measured in the study population. Similarly, correlation of serum levels of markers with PD effluent levels, which would reflect the local peritoneal milieu, was not possible due to lack of availability of the relevant data but is an area for further investigation.

Taken together, parameters (including those in the current study) based on serum and PD effluent biochemistry, along with additional available data on bone mineral health (such as DEXA scan), may be analysed using multivariate regression to possibly pave the way for the development of a validated risk stratification tool centred on severity of CKD-MDB in PD patients. This could be extremely valuable since such a model utilising information derived from non-invasive means (as opposed to diagnostic laparoscopy or peritoneal biopsy, for example) and readily available data points could inform the pre-emptive discontinuation of PD prior to EPS development, an area which is yet to be satisfactorily addressed despite substantial interest in the PD community for many years (4, 10). Furthermore, the predictive model could also benefit from input of real time independent variables, rather than binary single-time events such as presence of bloody ascites or bowel tethering on CT as outlined in other models (24).

Comparison of the relative efficacies of medical (phosphate binders, vitamin D analogues, calcimimetics) and surgical (parathyroidectomy) interventions in the prevention of EPS development in PD patients appears to be the next logical consideration. Unfortunately, subgroup analysis of the current cohort into the respective treatment categories would result in too small patient numbers to reliably answer this question. Importantly, analysis would be substantially confounded by patients crossing over into other treatment arms. Therefore, interrogation of larger multicentre data is required, with careful propensity matching of the allocated groups.

### EPS and the imbalance of pro-calcific and anti-calcific factors

In addition to the pro-calcific effects of increased tissue availability of phosphate and calcium, raised levels of ALP may potentially reduce the availability of calcification inhibitors, such as inorganic pyrophosphate (PP_i_) (32). Hydrolysis of PP_i_ also increases the tissue availability of phosphate causing further imbalance in the ratio of procalcifying factors and calcification inhibitors (33, 34). Uraemia is inherently a pro-inflammatory state and these patients are known to have low levels of Fetuin-A, another potent calcification inhibitor (31, 35). Peritoneal inflammation due to PD and episodes of peritonitis may further reduce the local availability of Fetuin-A (36) in the peritoneum, leading to an increased risk of peritoneal calcification. All of these factors could potentially lead to peritoneal calcification, either through direct precipitation or through a more likely mechanism of transition of mesothelial cells or myofibroblasts into osteoblasts (37). Of the latter route, Lansley and colleagues presented evidence of *in vitro* osteoblastic transformation of primary rat mesothelial cells when cultured in osteogenic media; significant upregulation of ALP expression and subsequent formation of mineralised bone-like nodules were reported (37). Interestingly, in human mesothelial cells exposed to osteogenic media, increases in mRNA expression of the osteoblast marker, RUNX2, varied between cells collected from different patients (37), suggesting that individual susceptibility to tissue calcification could be regulated on a cellular level. Thus, such individual factors outside of tangible endocrine diagnoses may contribute towards the pathoaetiology of EPS. Peritoneal calcification can contribute to tissue stiffening, which in turn can act as a trigger or ‘second hit’ leading to uncontrolled fibrosis (38) and EPS development. As an extension of this study, further efforts are therefore required to delineate the cellular and molecular mechanisms which regulate peritoneal calcification.

## Conclusion

In conclusion, these findings have demonstrated in a large study population that uncontrolled secondary hyperparathyroidism during peritoneal dialysis results in a 5- fold increased risk of subsequent EPS development. On a background of the known aetiological effects of peritoneal fibrosis or sclerosis due to PD, it appears that dysregulations in bone mineral metabolism may further contribute to the pathogenesis of EPS. Better management of secondary hyperparathyroidism during PD may help mitigate the risk of EPS development.

## Data availability statement

The raw data supporting the conclusions of this article will be made available by the authors, without undue reservation.

## Ethics statement

The studies involving humans were approved by Research and Development (R&D) department at Manchester University Hospital NHS Foundation Trust (MFT) (R04141). Research Ethics Committee review was not required under the Governance Arrangements for Research Ethics Committees (GAfREC) guidelines for research limited to the use of previously collected information. The studies were conducted in accordance with the local legislation and institutional requirements. The human samples used in this study were acquired from a by- product of routine care or industry. Written informed consent for participation was not required from the participants or the participants’ legal guardians/next of kin in accordance with the national legislation and institutional requirements.

## Author contributions

ZM: Conceptualization, Formal analysis, Investigation, Methodology, Writing – original draft. KW: Formal analysis, Writing – review & editing. CF: Formal Analysis, Software, Writing – review & editing. EW: Formal Analysis, Software, Writing – review & editing. AH: Supervision, Writing – review & editing. AV: Supervision, Writing – review & editing. SH: Investigation, Methodology, Supervision, Writing – review & editing. AS: Investigation, Writing – review & editing. TA: Supervision, Writing – review & editing. Dv: Conceptualization, Methodology, Supervision, Writing – review & editing.
